# CRISPRi-mediated *in vivo* gene silencing: a tool for prioritizing drug targets in *Mycobacterium abscessus*

**DOI:** 10.1128/aac.01889-25

**Published:** 2026-04-20

**Authors:** Rashmi Gupta, Breven S. Simcox, Kyle H. Rohde

**Affiliations:** 1 Division of Immunity and Pathogenesis, College of Medicine, Burnett School of Biomedical Sciences, University of Central Florida 6243, Orlando, Florida, USA; Queen Mary University of London, London, United Kingdom

**Keywords:** *Mycobacterium abscessus*, CRi, gene regulation, silencing, *ftsZ*

## Abstract

*Mycobacterium abscessus* (*Mab*) is a multidrug-resistant nontuberculous mycobacterium that causes debilitating tuberculosis (TB)-like pulmonary infections for which effective treatment options are lacking. One contributing factor to the poor *in vivo* efficacy of antibiotics may be the altered vulnerability of drug targets driven by host-specific environmental conditions. To enable validation and prioritization of candidate drug targets *in vivo*, we exploited CRISPRi (CRi) gene silencing in multiple mouse infection models. Inducible silencing of *ftsZ_Mab_*, a previously validated target, and three predicted targets (*leuS_Mab_*, *folP_Mab_*, and *fusA_Mab_*) confirmed their essentiality *in vitro*. We then assessed the *in vivo* vulnerability of these targets in both immunocompetent C57BL/6N and immunodeficient NOD SCID gamma (NSG) mice by evaluating the impact of CRi silencing on pulmonary mycobacterial burden. In NSG mice, silencing of each of these four genes led to comparable decreases in *Mab* burden. However, in C57BL/6N mice, the degree of *Mab* clearance varied among targets, suggesting that immune pressure may influence the outcome of CRi-mediated gene silencing. Notably, repression of *fusA_Mab_* yielded a larger decline in mycobacterial burden in C57BL/6N mice despite a lower level of gene silencing *in vitro,* consistent with the enhanced vulnerability of this target. Overall, this study demonstrated that *ftsZ_Mab_, leuS_Mab_*, *folP_Mab_*, and *fusA_Mab_* are essential for *Mab* growth *in vitro* and, for the first time, validated their vulnerability to inhibition by CRi during infection. These data also identified potential context-dependent target vulnerabilities, which could inform the prioritization of bacterial drug targets and accelerate the development of effective therapeutics for *Mab* infections.

## INTRODUCTION

The rising incidence of antibiotic resistance among bacterial pathogens poses a grave threat to global public health, necessitating the development of innovative therapeutic strategies. *Mycobacterium abscessus* (*Mab*), which causes chronic pulmonary infections, particularly in individuals with cystic fibrosis, chronic obstructive pulmonary disease (COPD), bronchiectasis, and other comorbidities (1–7), has emerged as an almost untreatable opportunistic pathogen. This is due to its intrinsic resistance to multiple antibiotics, including the ones used against tuberculosis (TB) infections, resulting from a highly impermeable cell wall, efficient efflux pump systems, and inducible resistance mechanisms that confer resistance to a broad spectrum of antibiotics (8–11). *Mab* infections require a prolonged, aggressive treatment regimen with multiple oral and injectable antibiotics, with failure rates that remain unacceptably high (up to 70%) (3, 12). In addition to the paucity of antibiotics able to effectively kill *Mab* even *in vitro,* discrepancies between *in vitro* and *in vivo* drug efficacy, which may be attributable to host-induced physiological changes in *Mab,* pose an additional challenge. This highlights the need to evaluate the vulnerability of candidate drug targets within the context of infection.

In recent years, CRISPR interference (CRi) has emerged as a powerful tool for gene manipulation, offering precise control over gene expression through transcriptional silencing of targeted genes. By utilizing a catalytically inactive variant of the CRISPR-associated protein Cas9 (dCas9) coupled with sequence-specific guide RNAs (sgRNAs), CRi enables efficient and reversible silencing of target genes upon induction with anhydrotetracycline (ATc) without inducing permanent genetic changes (13, 14). Several research groups, including ours, have utilized this tool in *Mab* to probe gene functions, including those essential for bacterial growth and drug resistance (15–19). CRi has numerous applications from studying genotype-phenotype relationships, targeting multiple genes to identify functional synthetic lethality, engineering metabolic pathways to optimize synergistic interactions, and identifying disease mechanisms (20–27). Applied to *M. tuberculosis* (*Mtb*)*,* CRi technology revealed the differential vulnerability of essential genes to silencing *in vitro*, which could serve as a proxy indicator of the vulnerability or “druggability” of drug targets (28). Evaluation of gene silencing *in vivo* will help us understand the physiological relevance and likely effectiveness of inhibitors of specific targets in a clinical context.

The combination of technologies like CRi with animal models provides a valuable strategy for assessing the impact of *in vivo* gene silencing on bacterial virulence, host-pathogen interactions, and potential therapeutic interventions. Several studies have focused on mycobacterial gene expression modulation in animals, primarily utilizing TetR-based systems that operate independently of CRi-mediated mechanism for regulating *Mtb* genes (29–36). However, the role of *Mab* genes, including virulence factors and potential drug targets *in vivo,* remains a significant knowledge gap due to delayed implementation of genetic tools in *Mab* and lack of animal models of persistent *Mab* infection (37, 38). To date, only a handful of studies involving silencing of *Mab* genes during infection have been reported. One study employed the Tet-OFF regulatory system to modulate the expression of the *mmpL3* mycolic acid transporter gene to confirm its essentiality in *Mab* during zebrafish infection (39). This animal model is evolutionarily distant from humans and thus lacks direct translational relevance to pathogenesis and infection. During the preparation of this manuscript, the reported application of CRi in more relevant murine models (40, 41) highlighted the potential of this approach to yield insights into the therapeutic potential of genes under physiological conditions. The ability to regulate gene expression during an infection would also provide the much-needed molecular tools to understand *Mab* pathogenesis and virulence.

Previously, we have utilized the mycobacterial CRi platform to validate essential genes and drug-resistance genes in *Mab* (16). In this study, we demonstrated CRi-mediated silencing of *ftsZ*, a previously validated target, during an animal infection using immunodeficient and immunocompetent mouse models. In addition, we validated three additional essential genes (*leuS_Mab_, fusA_Mab_, and folP_Mab_*) and evaluated their vulnerability to lethal silencing both *in vitro* and *in vivo*. Defining the differential vulnerability of essential genes, which represent candidate drug targets, to genetic silencing *in vivo* would be valuable toward validating and prioritizing new drug candidates for therapeutics development.

## RESULTS AND DISCUSSION

### CRi-mediated validation of predicted drug targets

**Fig 1:**
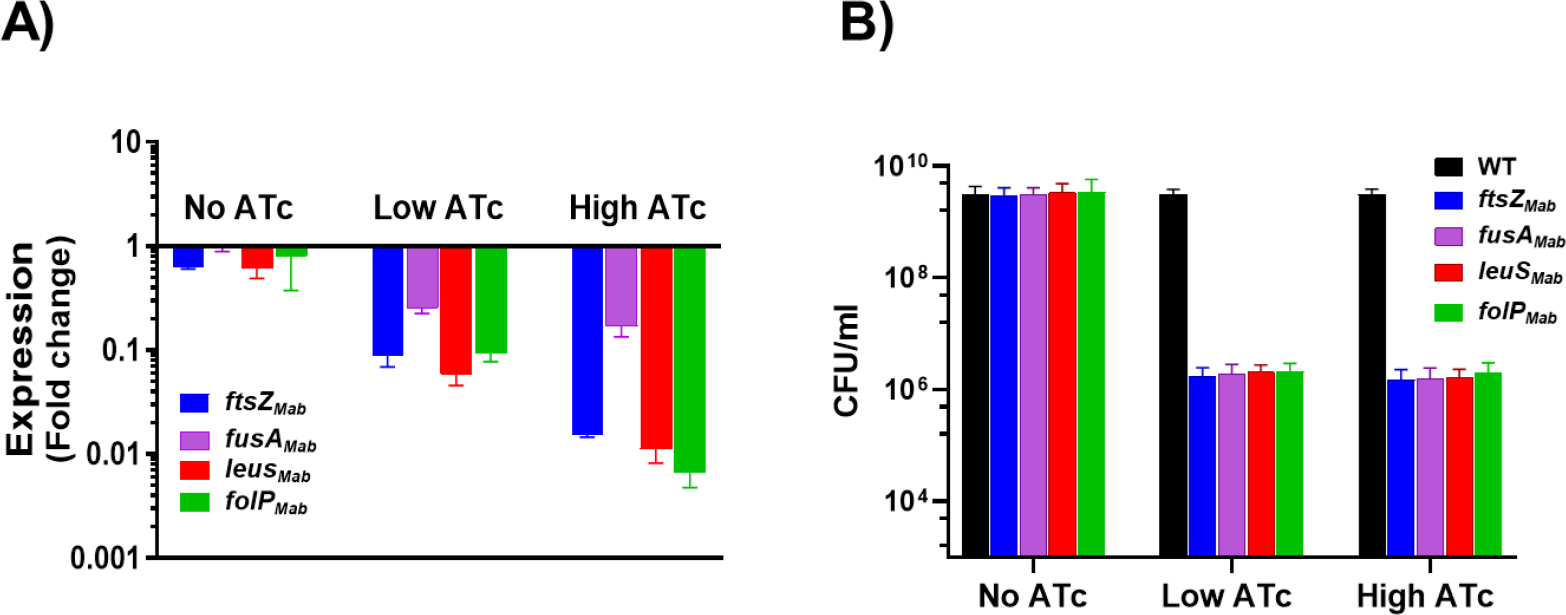
Impact of CRi silencing on predicted *Mab* essential genes. (**A**) mRNA levels relative to uninduced wild-type (WT) control. (**B**) CFU/mL upon ATc induction at low (200 ng/mL) and high (10 µg/mL) ATc concentrations; *N* = 3.

Previously, we utilized the mycobacterial single-plasmid pLJR962 CRi platform optimized for *M. smegmatis* featuring dCas9_Sth1_ (42) and demonstrated silencing and essentiality of *ftsZ_Mab_* (16). In this study, we evaluated the effect of CRi silencing of three additional genes with crucial roles in protein and nucleotide synthesis*—fusA_Mab,_* encoding elongation factor G (EF-G) (MAB_3849c); *leuS_Mab,_* coding for leucine tRNA synthetase (MAB_4923c); and *folP_Mab_*, a folate dihydropteroate synthase (MAB_0535). These targets are predicted to be essential based on Tn-seq analyses and corroborated by homology with essential *Mtb* genes as well as the antimicrobial efficacy of target-specific inhibitors against *Mtb* (43–48). We generated CRi constructs designed to hybridize adjacent to protospacer adjacent motifs (PAMs) with the highest predicted strength as described previously (Table S1) (16). After introduction into *Mab,* the impact of silencing on mRNA levels of the targeted gene and subsequent loss in cell viability (Fig. 1) was evaluated. Induction with either a low (200 ng/mL) or high dose (10 µg/mL) of ATc resulted in dose-dependent reduction in mRNA levels for all target genes, except for *fusA_Mab_* (Fig. 1A). At the low ATc dose, transcript levels for three of the target genes (*ftsZ_Mab_, leuS_Mab_, and folP_Mab_*) were reduced ~10-fold, whereas *fusA_Mab_* silencing had a lesser impact, with only ~4-fold decrease. The higher dose of ATc yielded ~100-fold reduction in *ftsZ_Mab_, leuS_Mab_,* and *folP_Mab_* transcripts, but no additional decline in *fusA_Mab_* mRNA levels was noted. Consistent with their predicted essentiality, transcriptional silencing of these genes led to a loss in bacterial viability following CRi induction. All the targets, including *fusA_Mab,_* exhibited a ~3–4 log decrease in colony-forming units (CFUs) (Fig. 1B; Fig. S1). The fact that ~20-fold less CRi silencing of *fusA_Mab_* resulted in bactericidal activity comparable to the other targets suggests that even partial knockdown is sufficient to disrupt the essential function of *fusA_Mab_*. This highlights the vulnerability of *fusA_Mab_*, supporting its prioritization as an optimal drug target. Overall, the strong correlation between transcriptional silencing and decreased *Mab* viability validates *leuS_Mab_, folP_Mab_,* and *fusA_Mab_* as essential genes and promising drug targets. However, their individual vulnerabilities may be significantly altered under disease-relevant conditions, making it crucial to assess the effect of gene silencing *in vivo*.

### *In vivo* CRi-mediated silencing in *Mab*

Studies implementing CRi silencing in two murine models were designed with two goals in mind: (i) demonstrating the feasibility of CRi-mediated gene silencing *in vivo* with simple ATc induction via drinking water and (ii) assessing whether immune pressure influences the vulnerability of different targets by comparing outcomes in immunocompetent C57BL/6N and immunodeficient NOD SCID gamma (NSG) mice. The presence or absence of an intact immune response in these models may alter the growth and physiology of *Mab* and/or impose stresses that affect the impact of silencing certain genes.

**Fig 2:**
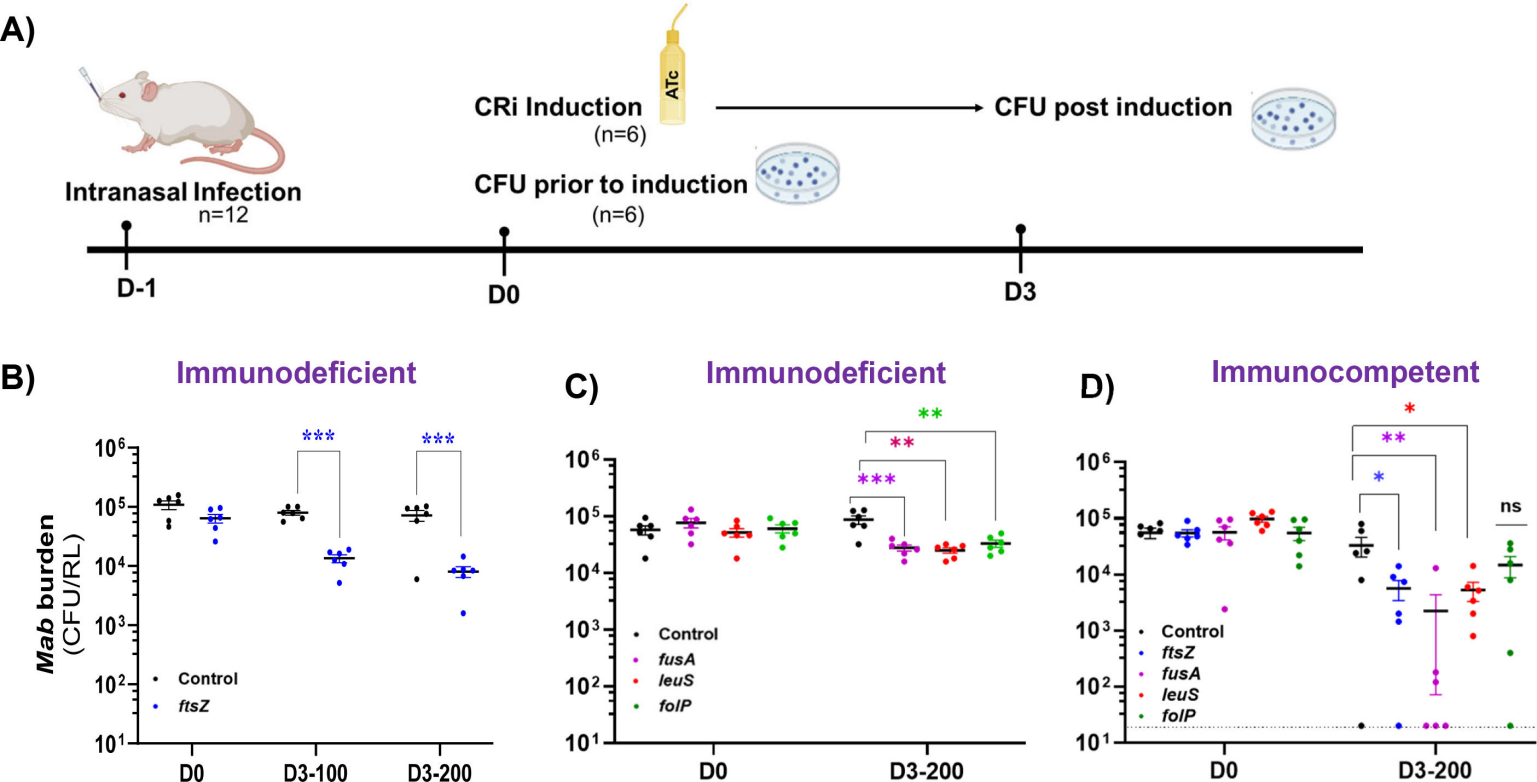
CRISPRi-mediated silencing of *Mab* genes in mice. (**A**) Illustration of the infection scheme. Mice were infected intranasally with control, *ftsZ_Mab,_* and other CRi strains (*fusA_Mab_, leuS_Mab_,* and *folP_Mab_*) and administered ATc in drinking water for 3 days. Prior to ATc induction at D0 (24 hpi) and at D3 (3d post-induction), mycobacterial burden in the right lung (RL) was determined. NSG mice induced with (**B**) 100 and 200 µg/mL ATc water and (**C**) with 200 µg/mL ATc water. (**D**) C57BL/6N mice induced with 200 µg/mL ATc water. Data are expressed as mean ±SEM of six mice per group per time point. Statistical analysis: (**B**) two-way ANOVA with Tukey’s multiple comparisons; (**C and D**) one-way ANOVA with Dunnett’s comparisons, *P ≤ 0.01, **P ≤ 0.001, and ***P ≤ 0.0001. Each dot represents one animal, and each figure represents independent experiments and conclusions drawn from within-batch comparisons.

Pulmonary infection with *Mab-*CRi strains was established via the intranasal route, as previously reported for *Mtb* and *M. bovis* (49, 50). Animals were infected with inoculum volumes of 40 µL based on the significantly enhanced infection efficiency (*P* < 0.005) compared to mice infected with a 20 µL volume containing the same total CFU (Fig. S2). Regardless of whether a supine or vertical position of mice was used during inoculum administration, infection with 40 µL of *Mab* yielded detectable CFU in the lungs of 100% of mice 24 h post-infection (hpi) versus a 78% infection rate when a smaller inoculum volume was used (Fig. S2). Initially, for proof-of-principle studies to test the feasibility of CRi-mediated gene silencing *in vivo*, we selected a previously validated essential target, *ftsZ_Mab_* (16). NOD SCID gamma (NSG) mice, which lack mature T cells, B cells, and functional natural killer (NK) cells, represent a simple model to study the effect of CRi gene silencing on *Mab* pulmonary burden without clearance mediated by innate and adaptive immunity. Immunodeficient NSG mice were infected with the *ftsZ_Mab_* CRi strain (or empty CRi vector) using the infection scheme outlined in Fig. 2A. Twenty-four hours post-infection, both the control and the *ftsZ* CRi groups of mice were provided drinking water supplemented with ATc inducer (100 and 200 µg/mL) and sucrose (to mask the taste of ATc and facilitate sufficient intake of ATc-laced water). Water intake records indicated a minimum of 3–4 mL water consumption by each mouse, which corresponds to approximately 600 µg ATc/mouse/day. Recent studies have demonstrated that ATc can mediate robust TetR-dependent gene repression in mice (40, 41, 51). Therefore, we used ATc to maintain consistency with *in vitro* studies while avoiding potential concerns about the antimicrobial effects of doxycycline, an alternative inducer with established pharmacokinetics and tissue distribution (31, 51, 52). When induced with 100 µg/mL ATc, the control group infected with *Mab* carrying the empty CRi vector maintained a stable bacterial burden, whereas CRi silencing of *ftsZ_Mab_* resulted in a 0.7-log decline relative to the control on day 3. Administration of 200 µg/mL ATc yielded a slightly higher 1-log decrease in CFU (Fig. 2B). Although the difference between the two doses was not statistically significant, the higher ATc dose was chosen for further studies based on the greater CFU decline noted. Thereafter, we infected NSG mice with other CRi strains (*leuS_Mab_*, *folP_Mab,_* and *fusA_Mab_*) and induced with 200 µg/mL ATc. Suppression of these three targets also resulted in a similar CFU decline (~0.5 log_10_), after 3 days of CRi induction by ATc (Fig. 2C). This short duration allowed ample time to observe a decline in *Mab* CFU for rapid assessment of target gene vulnerability, while also minimizing the emergence of CRi escape mutants, which we noted in previous *in vitro* studies (16).

Next, we sought to determine the impact of CRi silencing on *Mab* growth and survival during infection in immunocompetent mice to assess whether immune pressure or host-derived stresses could influence target vulnerability. In C57BL/6N mice, a ~0.8–1.0 log_10_ reduction in CFU was noted upon induction of silencing of three *Mab* gene targets (*ftsZ_Mab_*, *leuS_Mab_*, and *fusA_Mab_*)*,* whereas the *folP_Mab_* CRi strain showed no statistical decline (0.3 log_10_) in *Mab* burden compared to the control post-induction (D3) (Fig. 2D). This lesser effect of *folP_Mab_* CRi silencing in C57BL/6N relative to the other targets suggested target-specific vulnerability to inhibition dependent on the mouse strain. In contrast, transcriptional silencing of *fusA_Mab_* appeared to exert an enhanced lethal effect on *Mab* in this mouse background not observed in the immunodeficient NSG mouse model. While the average CFU of *Mab fusA_Mab_* CRi was only ~2-fold lower than those of *ftsZ_Mab_* and *leuS_Mab_* after CRi induction for 3 days, three of the six mice appeared to be sterilized below detection limits, of which two mice had ~2 log lower CFU compared to the single mouse with high burden (~10^4^ CFU). This indicates that genetic inhibition of *fusA_Mab,_* which serves as a proxy for antibiotic-mediated targeting of FusA, is more effective at clearing pulmonary *Mab* than inhibition of other targets. Interestingly, the increased vulnerability of *fusA_Mab_* was only noted in the context of an intact innate immune response. This is consistent with the high predicted vulnerability of *fusA_Mtb_* determined by Bosch et al. based on the rate of mycobacterial cell death upon induction of multiple CRi constructs targeting this gene (28, 43).

### Conclusion

*Mab* is a highly drug-resistant human pathogen, and existing treatments have a high failure rate, underscoring the need to identify new, effective treatment options. The identification and validation of new drug targets is one critical strategy to address this need. While high-throughput *in vitro* mycobacterial screens, such as a genome-wide gene expression (28)*,* and Tn-seq analysis (53) have identified essential targets and highlighted species-specific differences in gene essentiality, *in vivo* validation of target vulnerability remains crucial to determine physiological relevance and “druggability” in a clinically relevant context. CRi provides a versatile genetic tool for assessing the relative vulnerability of putative drug targets to inhibition, which facilitates prioritization of potential drug targets. Here, we extended our previous *in vitro* study (16) to silence predicted essential *Mab* genes *in vivo* to probe target vulnerabilities in the context of a pulmonary infection. The major findings of this study are as follows: (i) validation of the essential function of three additional *Mab* genes—*leuS_Mab_*, *folP_Mab,_* and *fusA_Mab_*, (ii) demonstration of CRi-mediated modulation of *Mab* gene expression in mice, and (iii) evidence for differences in gene vulnerabilities under *in vitro* versus *in vivo* conditions.

This study highlighted the feasibility of CRi-mediated *Mab* genes suppression *in vivo* in two distinct murine models, induced simply by addition of ATc in drinking water. The expression of selected gene targets was silenced by CRi induction after establishment of *Mab* infection to truly reflect gene silencing within the host environment. This is in contrast to a strategy implemented in recent studies in which target genes were preemptively silenced by ATc induction 3 days before infection (40, 41). This could have confounded the assessment of *in vivo* target vulnerability by depleting essential enzymes prior to infection and would have defeated the aim of silencing *Mab* genes *in vivo*. The NSG and C57BL6/N models, each characterized by distinct immune pressure, exhibited notable differences in respective vulnerabilities. A consistent silencing effect was noted in NSG mice with tight clustering of CFU in contrast to variable *Mab* clearance in C57BL/6N mice. These differences could be attributed to a combinatorial effect of the immune system and silencing by CRi. Comparison of the four essential targets investigated in this study indicated that *fusA_Mab_* was the most vulnerable to silencing both *in vitro* and *in vivo*. Despite much weaker CRi-mediated transcriptional repression of *fusA_Mab_*, the loss of viability *in vitro* was comparable to other targets. The target-specific vulnerability differences noted between immunocompetent and immunocompromised murine models suggest that immune pressure or other conditions within the host niche may alter the impact of target inhibition on mycobacterial viability. Whereas CRi silencing of *fusA_Mab_* in the context of an innate immune response caused a more dramatic reduction in CFU compared to *ftsZ_Mab_* or *leuS_Mab_,* silencing of *folP_Mab_* exhibited a dampened effect on *Mab* lung burden in immunocompetent mice. The discrepant outcomes of CRi gene silencing versus CFU loss *in vitro* or in NSG and C57BL/6N mice emphasize the importance of assessing drug target vulnerability in relevant mammalian *in vivo* models. We plan to evaluate additional potential drug targets in different immunocompetent mouse models, but this is beyond the scope of the current study. Application of this type of data to drug target selection would support the prioritization of *fusA_Mab_* over *folP_Mab_* based on the outsized effect on *Mab* viability compared to the level of gene silencing.

While this approach offers a rapid strategy to triage or rank drug target vulnerability *in vivo,* there are some caveats to be addressed in future studies. The short infection duration (3d) is not ideal to study targets associated with *Mab* persistence and adaptation. It was selected based on pilot studies indicating minimal added impact of longer ATc induction times on bacterial burden, perhaps due to the emergence of CRi escape mutants observed in our previous *in vitro* study (16). A detailed evaluation of the extent of this problem during *in vivo* CRi assays and mitigation strategies are important next steps in the development of this technology. The lack of an immunocompetent mouse model of chronic *Mab* infection also limits the *in vivo* application of CRi to study drug targets and virulence mechanisms under conditions that better mimic natural infections. Efforts are ongoing in our lab and others to address this need.

In conclusion, we have demonstrated a simple method for CRi-mediated *Mab* gene silencing during host infection useful for rapidly assessing the vulnerability of candidate drug targets to inform target prioritization. We have also validated the essentiality of four putative drug targets both *in vitro* and *in vivo,* providing the premise for developing antibiotics targeting these essential mycobacterial enzymes. Consistent with observations in *Mtb,* the translation elongation factor FusA appears to be a highly vulnerable target in *Mab,* which requires minimal perturbation to yield bactericidal outcomes. If validated by further studies, the hint that inhibition of some targets, but not others, may synergize with immune mediators to clear *Mab* could be leveraged as an additional criterion for target prioritization. The ability to simultaneously silence multiple genes using CRi affords a way to genetically explore potential synergy or antagonism between drug targets *in vivo* to aid the design of multidrug regimens.

## MATERIALS AND METHODS

### Bacterial strains and culture conditions

A smooth strain of *Mab* 390S (54) and other *Mab*-CRi strains used in this study (Table 1) were cultured in Middlebrook 7H9 supplemented with 0.05% Tween 80 and 10% oleic acid/albumin/dextrose/catalase (OADC) and incubated at 37°C and 5% CO_2_. Kanamycin 50 µg/mL (KAN_50_), apramycin 50 µg/mL (APRA_50_), cycloheximide 100 µg/mL, and amikacin 32 µg/mL (AMK_32_) were added when appropriate, depending on the expected resistance profile of the strain. A stock solution of anhydrotetracycline (ATc) was prepared according to the manufacturer’s instructions. Stocks were stored at −20°C.

**TABLE 1:** Bacterial plasmids and strains

Name	Genotype/phenotype	Reference
Plasmids		
pLJR962	Mycobacterial CRISPRi vector optimized for *M. smegmatis*	Addgene plasmid # 115162
pLJR962-FtsZ	CRISPRi construct with 20 nt sgRNA of *ftsZ_Mab_* in plasmid pLJR962	(16)
pLJR962-LeuS	CRISPRi construct with 20 nt sgRNA of *leuS_Mab_* in plasmid pLJR962	This study
pLJR962-FolP	CRISPRi construct with 21 nt sgRNA of *folP_Mab_* in plasmid pLJR962	This study
pLJR962-FusA	CRISPRi construct with 20 nt sgRNA of *fusA_Mab_* in plasmid pLJR962	This study
Strains		
Mab390S Mab_CRi_pLJR962	Smooth strain, *Mycobacterium abscessus* 390S WT transformed pLJR962 plasmid with no target sgRNA	(54) (16)
Mab_ftsZ_CRISPRi_pLJR962	390S WT transformed with pLJR962-FtsZ plasmid	(16)
Mab_leuS_CRISPRi_pLJR962	390S WT transformed with pLJR962-LeuS plasmid	This study
Mab_folP_CRISPRi_pLJR962	390S WT transformed with pLJR962-FolP plasmid	This study
Mab_fusA_CRISPRi_pLJR962	390S WT transformed with pLJR965-FusA plasmid	This study

### Construction of *Mab* CRi knockdowns

We constructed CRi knockdowns in four genes (MAB_0535: dihydropterate synthase, *folP*_*Mab*_; MAB_4923c: leucine tRNA ligase, *leuS*_*Mab*_; MAB_2009: cell division protein, *ftsZ_Mab_*; and MAB_3849c: elongation factor G, *fusA_Mab_*) in pLJR962 (a gift from Sarah Fortune, Addgene plasmids #115162 and #11563, http://n2t.net/addgene:115162, http://n2t.net/addgene:115163) (42). PAM and sgRNA target sequence selection was carried out as per Rock et al.’s study (42). CRi constructs of *leuS*_*Mab*_, *folP*_*Mab*_, *ftsZ*_*Mab*_, and *fusA_Mab_* were created using a PCR-based mutagenesis strategy, as described earlier (16). See Table S2 for sequences of primers used for CRi plasmid generation, colony screening, and sequencing.

### RNA isolation and qRT-PCR

*Mab* CRi strains were grown and induced by ATc for 20 h, as described before (16). An uninduced sample was kept as the control. RNA isolation was carried out using the RNeasy kit, as described previously (16, 55). Ct values were normalized against the housekeeping gene, *sigA,* and fold change was calculated against an uninduced sample run in parallel using the −2^ΔΔCt^ method. Fold change is represented relative to WT uninduced (no ATc). All qRT-PCR primers are listed in Table S2.

### CFU enumeration

A dilution-plating assay was conducted to determine the effect of CRi-mediated repression of the putative essential genes, *ftsZ_Mab_*, *leuS_Mab_*, *folP_Mab,_* and *fusA_Mab_* on cell viability. For the assay, the cultures were grown from freezer stock to log phase (OD_600 =_0.4–0.8), then diluted back to OD_600 =_0.3, and 5 µL of 2-fold serial dilutions was spotted onto 7H10 agar plates with and without ATc. The plates were incubated at 37°C for 5 days, and CFU/mL was calculated.

### *In vivo* CRi silencing

We utilized immunocompetent C57BL6/N and immunodeficient NOD scid gamma mouse (NSG) murine models to assess *Mab* gene silencing during murine infection. C57BL6/N (Taconic Biosciences) and NOD SCID gamma (NSG) mice (UCF breeding colony) were housed in an AAALAC-accredited UCF Lake Nona vivarium under stringent sterile conditions. Animal infections were carried out according to an approved IACUC protocol (IPROTO202300044). Male and female mice (8 to 10 weeks old) were anesthetized with inhaled isoflurane (2.5 L/min) and intranasally infected with an inoculum of 10^5^ CFU of five CRi-*Mab* strains, including the control (empty vector) strain. A log-phase culture was syringed 5× using a 27-G needle syringe to disrupt clumps before dilution in 40 µL PBS. Each group consisted of six mice per time point with an equal sex distribution (three males and three females). After 24 h of infection, animals were euthanized to assess baseline infection levels, which served as D0 controls. Thereafter, mice, including those which received the empty vector, were treated with ATc-supplemented (at the indicated concentration) and sucrose (5%)-supplemented drinking water in red-tinted water bottles (light protection). After 3 days of ATc induction, lungs were harvested to determine the mycobacterial burden. Right lungs were excised and homogenized in 1 mL PBS with 1-mm-diameter silicon carbide beads (0.5 mL) in a bead beater (Biospec) for 2 min (maximum speed). The homogenized lung tissue was then serially diluted and plated (50 µl) onto 7H10 agar plates. After 5–7 days of incubation, colonies were counted, and CFU/mL was calculated. In samples where colonies could not be recovered, the lower limit of detection (LoD) of 20 CFU was accounted in statistical analysis.

### Statistical analysis

A statistical difference was determined using *t*-tests with pairwise comparisons and ANOVA analyses with appropriate Tukey or Dunnett’s post test analysis. GraphPad Prism was used for this analysis. Each data point indicates a single animal unless otherwise stated. Statistical tests are indicated in the legends wherever appropriate.

## SUPPLEMENTAL MATERIAL

Supplemental figures aac.01889-25-s0001.pdf Fig. S1 and S2.

Supplemental tables aac.01889-25-s0002.pdf Tables S1 and S2.

ASM does not own the copyrights to Supplemental Material that may be linked to, or accessed through, an article. The authors have granted ASM a non-exclusive, world-wide license to publish the Supplemental Material files. Please contact the corresponding author directly for reuse.
