# Supplementary material for: CRISPRi-mediated in vivo gene silencing: a tool for prioritizing drug targets in Mycobacterium abscessus
Source: Antimicrob Agents Chemother. 2026 Apr 20;70(6):e01889-25. doi: 10.1128/aac.01889-25 (PMC13231894; doi:10.1128/aac.01889-25)
Supplement: Supplemental figures — Fig. S1 and S2. [file aac.01889-25-s0001.pdf]

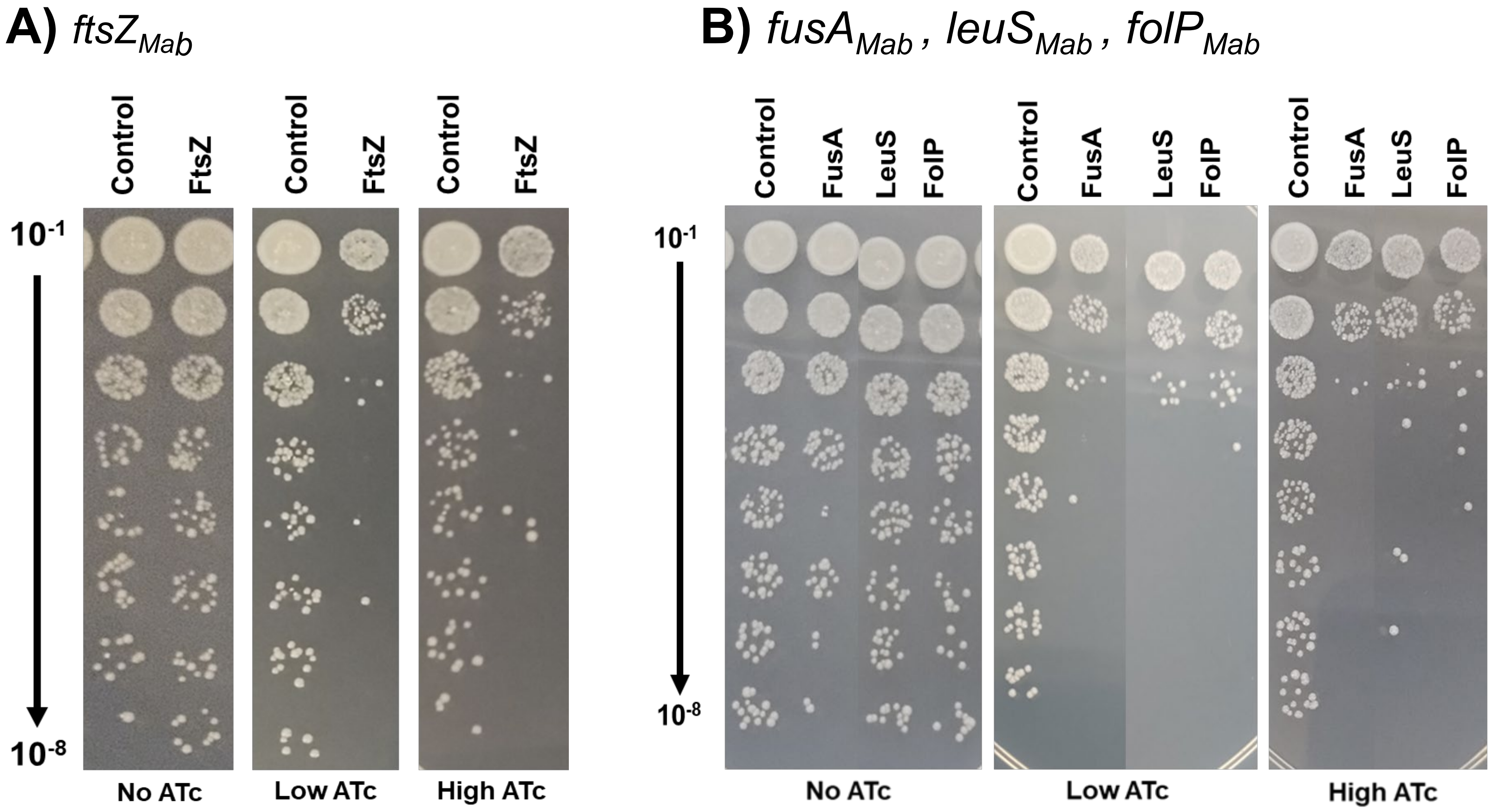

Figure S1: CRi silencing of *Mab* potential drug targets. *Mab* growth upon ATc induction at low (200ng/ml) and high (10µg/ml) inducer concentration. **(A)** *ftsZ*$_{Mab}$ and **(B)** *fusA*$_{Mab}$, *leuS*$_{Mab}$, *folP*$_{Mab}$ gene targets. This is a representative image compiled from different plates from the same experiment.

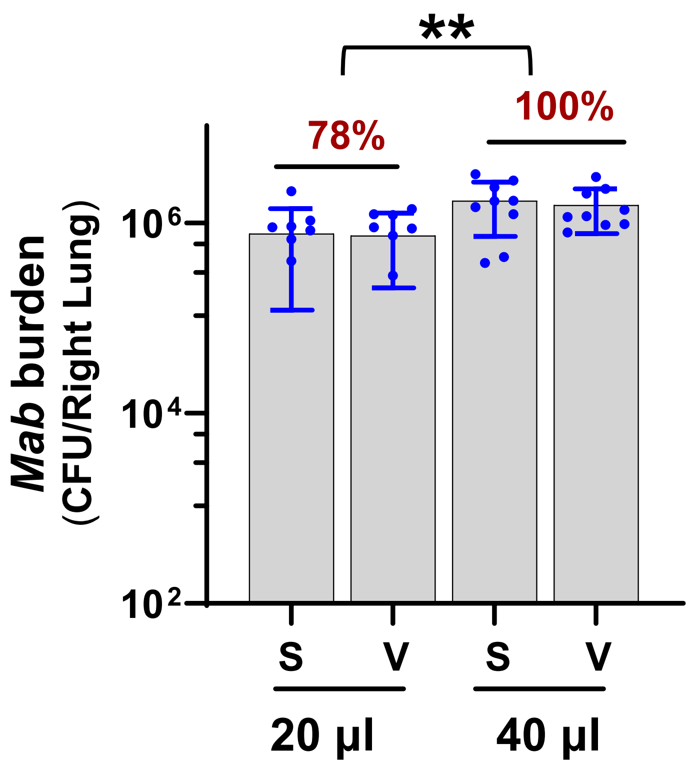

Figure S2: **Impact of dosing volume and body position on *Mab* infection efficiency.** *Mab* load in the lungs of infected C57BL/6N mice when infected with 10^6 CFU at indicated dosing volumes when held in supine (S) or vertical (V) position. The data is from a total of 9 animals per group from 3 pooled experiments. ** P=0.001, unpaired t-tests with Welch's correction. 78% indicates the infection efficiency where colonies were only recovered from the lungs of 14 out of 18 mice whereas 100% indicate *Mab* recovery from all 18 animals. Each dot represents one animal.
