# Supplementary material for: CRISPRi-mediated in vivo gene silencing: a tool for prioritizing drug targets in Mycobacterium abscessus
Source: Antimicrob Agents Chemother. 2026 Apr 20;70(6):e01889-25. doi: 10.1128/aac.01889-25 (PMC13231894; doi:10.1128/aac.01889-25)
Supplement: Supplemental tables — Tables S1 and S2. [file aac.01889-25-s0002.pdf]

Table S1: Potential *Mab* Drug Targets

Cell Process	Target ID	Target Name	PAM seq (5'-3'), fold-repression[#]	Target sequence (5' to 3')	Location from start (bp)
**Cell Division**	MAB_2009	FtsZ (Cell division)	GTTCT,120.5	ttcgagggcaagcggcgcagc	403
**Protein Synthesis**	MAB_3849c	FusA (Elongation factor G)	CTTCT, 216.7	aacggcaaccagatcaacat	223
	MAB_4923c	LeuS (Leucine tRNA ligase)	CTTCT, 216.7	aagtggacgcagtggatctt	487
**Folate Metabolism**	MAB_0535	FolP (Dihydropteroate synthase)	CTTCT, 216.7	gacggtggacgattcgtccgt	79

[#] Fold repression reported in *M. smegmatis* (Rock *et al.* Ref 43)

Table S2 : CRISPRi primers

Purpose	Primer Name	Sequence (5'- 3')	Comments
**Cloning**			
leuS $_{Mab}$	CRiMab_LeuS_F	**AAGATCCACTGCGTCCACTT**GTTTTTGTACTCGAAAGAAGCTACAA	Forward cloning primer
folP $_{Mab}$	CRiMab_FolP_F	**ACGGACGAATCGTCCACCGTC**GTTTTTGTACTCGAAAGAAGCTACAA	Forward cloning primer
fusA $_{Mab}$	CRiMab_FusA_F	**ATGTTGATCTGGTTGCCGTT**GTTTTTGTACTCGAAAGAAGCTACAA	Forward cloning primer
For all single CRi-constructs	CRi_R	CTCCCAGATTATATCTATCACTGATAGGGATCG	Universal reverse cloning primer
**PCR Colony Screening**			
	CRiMab_LeuS_scr_F	AAGATCCACTGCGTCCAC	Forward sequencing Primer
	CRiMab_FolP_scr_F	ACGGACGAATCGTCCAC	Forward sequencing Primer
	CRiMab_FusA_scr_F	ATGTTGATCTGGTTGCCGTTGTTTTTG	Forward sequencing Primer
	Mab_CRi-seq_R	GAGCCATTGATAATGCTCTTCATCC	Universal reverse primer
**Sequencing**			
	Mab_CRi-seq_R	GAGCCATTGATAATGCTCTTCATCC	Universal reverse primer
**qRT-PCR**			
	LeuS$_{Mab}$_qRT-F	CGCGCATCGAATACAAACTG	Forward RT-PCR primer
	LeuS$_{Mab}$_qRT-R	GGGCAATTCGACGGGTAATAC	Reverse RT-PCR primer
	FusA$_{Mab}$_qRT-F	GTGCTTACCGACCTGAACAAG	Forward RT-PCR primer
	FusA$_{Mab}$_qRT-R	GGGTTTCACCGATCTTGTAGTTC	Reverse RT-PCR primer
	FolP$_{Mab}$_qRT-F	ACGACTCCTTCTCTGAC	Forward RT-PCR primer
	FolP$_{Mab}$_qRT-R	TCGATCTTCTGCCTGAT	Reverse RT-PCR primer

Bold:specific gene sgRNA insert sequence
